# Supplementary material for: Ongoing lymphoid HIV production drives pyroptosis and GLP-1 counter-regulation in ART-suppressed infection
Source: bioRxiv. 2026 Jan 10:2026.01.09.698696. Preprint. [Version 1] doi: 10.64898/2026.01.09.698696 (PMC12803172; doi:10.64898/2026.01.09.698696)
Supplement: Supplement 1 [file media-1.pdf]

SUPPLEMENTARY MATERIAL
Ongoing lymphoid HIV production drives pyroptosis and GLP-1 counter-regulation in ART-suppressed infection

Supplementary Table 1. r^2 and p values for the association between $vRNA^+$ cells in LNs and plasma cytokines that were not statistically significant.

Cytokine or biomarker (plasma)	HIV RNA^+ cells in LNs	
	r^2	p
TNF-α	0.006	0.758
MIP-1β	0.984	0.205
IL-23	0.019	0.581
IL-2	0.002	0.844
IL-17	0.012	0.669
IL-12p70	0.000	0.947
IFN-γ	0.008	0.718
IL-1β	0.030	0.534
IL-10	0.129	0.156
IL-6	0.023	0.533
IL-18	0.004	0.791

Supplementary Table 2. Relationship between GLP-1^+ ileal cells and plasma cytokines. The r^2 and p values are derived from linear regression.

Plasma cytokine	GLP-1^+ ileal cells	
	r	p
IL-10*	0.387	0.004
IL-1β*	0.421	0.003
IL-6*	0.369	0.006
IL-18*	0.293	0.02
IL-5	0.206	0.05
IL-8*	0.207	0.05
IL-23	0.182	0.07
IL-2	0.166	0.08
IL-17A*	0.157	0.09
IL-7	0.143	0.11
IL-12p70	0.121	0.14
IFN-γ	0.109	0.17
MIP-1β	0.006	0.75
TNF-α*	0.00	0.95

*Cytokines associated with pyroptosis.

Supplementary Table 3. Antibodies used for IHC.

Antibody	Company	Catalog number	Dilution
GLP-1	Abcam	ab108443	1:100
GSD	Cell Signaling	36425	1:200
IL-6	Proteintech	21865-1-AP	1:1500
CD68	Biocare Medical	CM033	1:400
IL-18	Abcam	ab191152	1:100
